# The genera *Albanura* Deharveng, 1982 and *Persanura* Mayvan et al., 2015 are no longer monotypic: description of new species from the Caucasus (Collembola, Neanuridae, Neanurinae, Neanurini)

**DOI:** 10.3897/zookeys.737.21191

**Published:** 2018-02-12

**Authors:** Adrian Smolis, Nataliya Kuznetsova

**Affiliations:** 1 Institute of Environmental Biology, Department of Invertebrate Biology, Evolution and Conservation, University of Wrocław, Przybyszewskiego 65, 51-148 Wrocław, Poland; 2 Institute of Biology and Chemistry, Moscow State Pedagogical University, Moscow 129164, Russia

**Keywords:** Azerbaijan, Russia, taxonomy

## Abstract

Two new species from the Caucasus belonging to the genera *Albanura* and *Persanura* are described and illustrated in detail. *Albanura secunda* **sp. n.** is distinctive because of the presence of chaetae E on the head as well as three ordinary chaetae on tubercles De of thorax III and abdomen I–III. Additionally, the species can be recognized by the absence of chaeta O on the head and presence of 3+3 chaetae Di on abdomen V. The most important characters that can be used to distinguish *Persanura lencarana* **sp. n.** are the labral formula, an increased number of chaetae De on thorax II and III, and the number of chaetae Di on the thorax and abdomen V. Comments on the status of the genera and the affinities of the Caucasian fauna of Neanurinae are also given.

## Introduction

Creating and establishing new genera, especially monotypic ones, may be very problematic and complicated for taxonomists. Very often such decisions are questioned by other specialists, and authors who decide on such choices are often labelled as ‘splitters’. Interestingly, the term ‘splitter’ was used for the first time by one of the most famous biologists, Charles Robert Darwin. Among Neanurini, one of the six tribes within the subfamily Neanurinae (Cassagnau, 1989), there are both large and “megadiverse” genera, e.g., *Deutonura* Cassagnau, 1979 and *Endonura* Cassagnau, 1979 comprising 57 and 51 valid species, respectively (Cassagnau 1979, Smolis and Kaprus’ 2009, Porco et al. 2010, Smolis et al. 2011, Deharveng et al. 2015, Smolis et al. 2016, Smolis and Kuznetsova 2016, Smolis et al. 2017), and several monotypic ones, e.g. *Albanura* Deharveng, 1982, *Cansilianura* Dallai & Fanciulli, 1983, *Edoughnura* Deharveng et al., 2007, *Xylanura* Smolis, 2011, or *Persanura* Mayvan et al., 2015 (Deharveng 1982, Dallai and Fanciulli 1983, Deharveng et al. 2007, Smolis 2011, Mayvan et al. 2015).

The examination of rich Neanurinae materials from the Caucasus collected during primarily ecological research has revealed two unknown species that should be classified within the so far monotypic genera *Albanura* and *Persanura*. The genus *Albanura* was created by Deharveng (1982) for Neanura (Deutonura) nana Cassagnau & Péja, 1979, from Albania (Cassagnau and Péja 1979), whilst the genus *Persanura* was established recently by Mayvan et al. (2015) for the species *Persanura hyrcanica* Mayvan et al., 2015 from Iran. Illustrated descriptions of the new species with remarks on the status of both corresponding genera and the possible origin of the Neanurinae fauna of the region are provided herewith.

## Materials and methods

The specimens were cleared in Nesbitt’s fluid, subsequently mounted on slides in Phoera liquid (200 g of chloral hydrate, 30 g of arabic gum and 20 g of glycerol dissolved and mixed into 50 g of distilled water) and studied using a Nikon Eclipse E600 phase contrast microscope. Figures were drawn with camera lucida and prepared for publication using Adobe Photoshop CS3.

Institutions of depository of materials:

**DIBEC** Department of Invertebrate Biology, Evolution and Conservation, Institute of Environmental Biology, University of Wrocław, Poland

**MSPU** Moscow State Pedagogical University, Institute of Biology and Chemistry, Moscow, Russia

**Terminology.** The terminology and abbreviations used in the paper are those of Deharveng (1983), Deharveng and Weiner (1984), Smolis and Deharveng (2006) and Smolis (2008).

**Abbreviations used.** General morphology: **Abd**. – Abdomen, **Ant**. – antenna, **AOIII** – sensory organ of antennal segment III, **Cx** – coxa, **Fe** – femur, **Scx2** – subcoxa 2, **T** – tibiotarsus, **Th**. – thorax, **Tr** – trochanter, **VT** – ventral tube.

Groups of chaetae: **Ag** – antegenital, **An** – chaetae of anal lobes, **ap** – apical, **ca** – centroapical, **cm** – centromedial, **cp** – centroposterior, **d** – dorsal, **Fu** – furcal, **vc** – ventrocentral, **Ve** or **ve** – ventroexternal, **Vea** – ventroexternoanterior, **Vem** – ventroexternomedial, **Vep** – ventroexteroposterior, **Vel** – ventroexternolateral, **Vec** – ventroexternocentral, **Vei** – ventroexternointernal, **Vi** or **vi** – ventrointernal, **Vl** – ventrolateral.

Tubercles: **Af** – antenno-frontal, **Cl** – clypeal, **De** – dorsoexternal, **Di** – dorsointernal, **Dl** – dorsolateral, **L** – lateral, **Oc** – ocular, **So** – subocular.

Types of chaetae: **Ml** – long macrochaeta, **Mc** – short macrochaeta, **Mcc** – very short macrochaeta, **me** – mesochaeta, **mi** – microchaeta, **ms** – s-microchaeta, **S** or **s** – chaeta s, **bs** – s-chaeta on Ant. IV, **miA** – microchaetae on Ant. IV, **iv** – ordinary chaetae on ventral Ant. IV, **or** – organite of Ant IV, **brs** – border s-chaeta on Ant. IV, **i** – ordinary chaeta on Ant. IV, **mou** – cylindrical s-chaetae on Ant IV („soies mousses”), **x** – labial papilla x, **L**’ – ordinary lateral chaeta on Abd. V, **B4**, **B5** – ordinary chaetae on tibiotarsi.

## Taxonomy

### Albanura secunda sp. n.

Taxon classificationAnimaliaCollembolaNeanuridae

http://zoobank.org/BC401778-36A5-4296-9AD9-AD0C74C291CF

Figs 1–13 , Tables 1 , 2 , 3

#### Type material.

Holotype: male on slide, Russia, Caucasus,Karachaevo-Cherkessiya, Teberdinsky Reserve, spruce forest, litter, Oct.1978, leg. E. Dobrolyubova (DIBEC). Paratype: female on slide, same data as for holotype.

#### Etymology.

The name *secunda* refers to the fact that the new species is the second member of the genus.

#### Diagnosis.

Habitus similar to that of *Albanura nana*. Dorsal tubercles present and well developed. Buccal cone long, labrum ogival. Head with chaetae A, B, C, D and E. Chaeta O absent. Tubercles Dl and (L+So) on head with six and nine chaetae respectively. Tubercles De on Th. II and III with three and four chaetae respectively. Tubercles De on Abd. I–III with four chaetae. Tubercles L on Abd. III and IV with four and seven chaetae respectively. Abd. IV and V with seven and three tubercles respectively. Abd. V with 3+3 chaetae Di.

#### Description.

**Figures 1–13.**
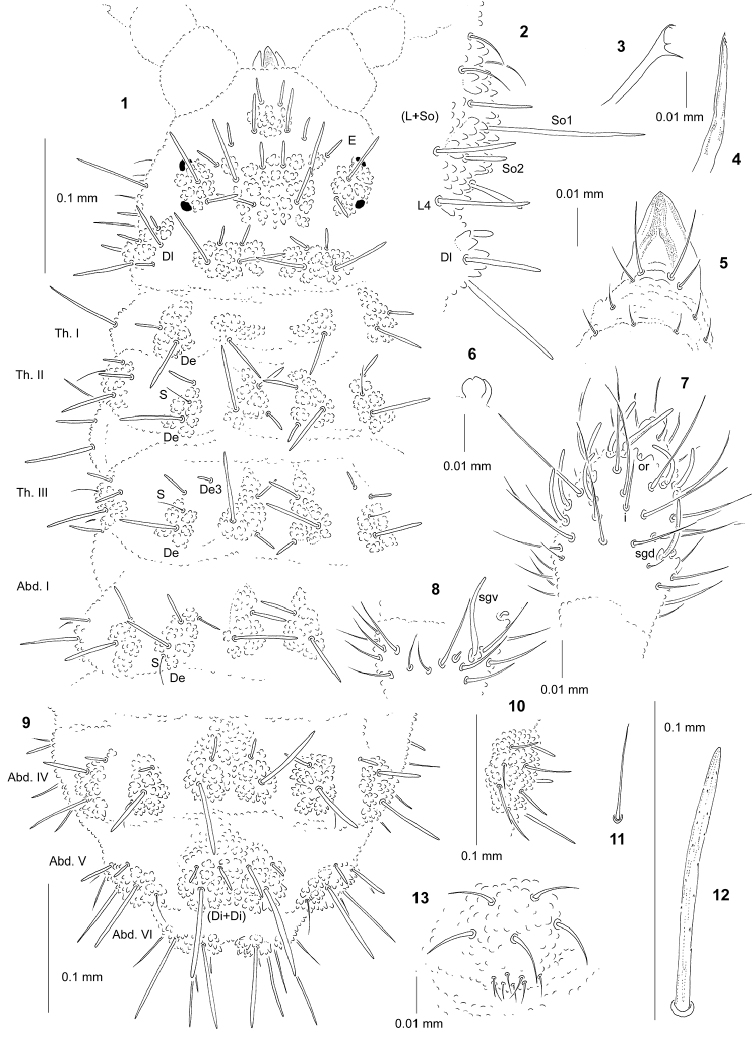
*Albanura secunda* sp. n.: **1** chaetotaxy of head, Th. and Abd. I (holotype), dorsal view **2** chaetotaxy of tubercles Dl and (L+So) on head, ventrolateral view **3** mandible **4** maxilla **5** chaetotaxy and ventral sclerifications of labrum **6** apical bulb, ventral view **7** dorsal chaetotaxy of Ant. III–IV **8** ventral chaetotaxy of Ant. III **9** dorsal chaetotaxy of Abd. IV–VI **10** tubercle L of Abd. IV **11** sensillum of Abd. V **12** chaeta Di1 of Abd. V **13** furca rudimentary.

General. Body length (without antennae): 0.8 mm (holotype), 0.9 mm (paratype). Colour of the body white. 2+2 rather large black eyes, in a typical arrangement for the genus (one anterior and one posterior, Fig. 1).

***Chaetal morphology.*** Dorsal ordinary chaetae of five types: long macrochaetae, short macrochaetae, very short macrochaetae, mesochaetae and microchaetae. Long macrochaetae relatively thick and short, straight, narrowly sheathed, feebly serrated, apically arc-like or rounded (Figs 1, 9, 12). Macrochaetae Mc and Mcc morphologically similar to long macrochaetae, but much shorter. Mesochaetae similar to ventral chaetae, thin, smooth and pointed. Microchaetae similar to mesochaetae, but shorter. S-chaetae of tergites thin, smooth and short, distinctly shorter than nearby macrochaetae (Figs 1, 9, 11).

***Antennae.*** Typical of the genus. Dorsal chaetotaxy of Ant. III–IV as in Fig. 7 and Table 2. S-chaetae of Ant. IV of medium length and moderately thickened (Fig. 7). Apical vesicle distinct and trilobed (Fig. 6). Ventral chaetotaxy of Ant. III–IV as in Fig. 8 and Table 2.

***Mouthparts.*** Buccal cone long with labral sclerifications ogival. Labrum chaetotaxy: 4/2, 4 (Fig. 5). Labium with four basal, three distal and four lateral chaetae, papillae x absent. Maxilla styliform, mandible thin and tridentate (Figs 3, 4).

***Dorsal chaetotaxy and tubercles.*** Chaetotaxy of head as in Figs 1, 2 and Table 1. Chaetotaxy of thorax and abdomen as in Figs 3, 4 and Table 3. Th. III with chaetae De3 free (Fig. 1). On Abd. I–III, the line of chaetae De1–chaeta s is not perpendicular to the dorsomedian line. No cryptopygy, Abd. VI well visible from above (Fig. 9).

**Table 1.** Chaetotaxy of *Albanura secunda* sp. n.: cephalic chaetotaxy, dorsal side.

Tubercle	Number of chaetae	Types of chaetae	Names of chaetae
Cl	4	Mc	F
		Mcc	G
Af	10	Ml	B
		Mc	A
		Mcc	C, D, E
Oc	3	Ml	Ocm
		Mcc	Ocp
		mi	Oca
(Di+De)	4	Ml	Di1, De1
		Mcc	Di2, De2
Dl	6	Ml	Dl5, Dl1
		Mc	Dl3, Dl4
		Mcc	Dl2, Dl6
(L+So)	8	Ml	So1
		Mc	L1, L2, L4
		Mcc	So2, So6
		me	So3–5

**Table 2.** Chaetotaxy of *Albanura secunda* sp. n.: chaetotaxy of antennae.

Segment, Group	Number of chaetae	Segment, Group	Number of chaetae adult
I	7	IV	or, 8 S, i, 12 mou, 6 brs, 2 iv
II	12		
III	5 sensilla AO III		
ve	5	ap	8 bs, 5 miA
vc	4	ca	2 bs, 3 miA
vi	4	cm	3 bs, 1 miA
d	5	cp	8 miA, 1 brs

**Table 3.** Chaetotaxy of *Albanura secunda* sp. n.: postcephalic chaetotaxy.

Terga					Legs				
	Di	De	Dl	L	Scx2	Cx	Tr	Fe	T
Th. I	1	2	1	-	0	3	6	13	19
Th. II	3	2+s	3+s+ms	3	2	7	6	12	19
Th. III	3	3+s	3+s	3	2	8	6	11	18
					Sterna				
Abd. I	2	3+s	2	3	VT: 4				
Abd. II	2	3+s	2	3	Ve: 5; chaeta Ve1 present				
Abd. III	2	3+s	2	4	Vel:5; Fu: 5 me, 8 mi				
Abd. IV	(2+2)	2+s	3	7	Vel: 4; Vec: 2; Vei: 2; Vl: 4				
Abd. V	(3+3)	7+s			Ag: 3; Vl: 1, L‘: 1				
Abd. VI		7			Ve: 13–14; An: 2mi				

***Ventral chaetotaxy.*** On head, groups Vea, Vem and Vep with 3, 3, 4 chaetae respectively. Group Vi on head with 6 chaetae. Group L on Abd. IV with 7 chaetae (Fig. 10). On Abd. IV, furca rudimentary with 8 microchaetae (Fig. 13). On Abd. V, chaetae Vl and L’ present.

***Legs.*** Chaetotaxy of legs as in Table 3. Claw without internal tooth. On tibiotarsus, chaeta M present and chaetae B4 and B5 relatively short and pointed.

#### Remarks.

*Albanura secunda* sp. n is easily distinguished from *A. nana* Cassagnau & Péja, 1979, the only other known species in the genus, by its dorsal chaetotaxy: presence/absence of chaeta O on the head (absent in *secunda*; present in *nana*), presence/absence of chaetae E on the head (present in *secunda*; absent in *nana*), number of chaetae Dl on the head (six in *secunda*; five in *nana*), number of ordinary chaetae De on Th. III (three in *secunda*; two in *nana*), number of ordinary chaetae De on Abd. I–III (three in *secunda*; two in *nana*), number of chaetae L on Abd. IV (seven in *secunda*; five in *nana*), and number of chaetae Di on Abd. V (3+3 in *secunda*; 2+2 in *nana*).

### Persanura lencarana sp. n.

Taxon classificationAnimaliaCollembolaNeanuridae

http://zoobank.org/9779E436-87E7-4FBA-8B7E-359701EC0655

Figs 14–28 , Table 4 , 5 , 6

#### Type material.

Holotype: female on slide, Azerbaijan, Lankaran District, surroundings of Dashdatuk, forest, under stones, 30.I.1985, leg. Expedition of Moscow Pedagogical State University (MSPU).

#### Etymology.

The name “*lencarana*” refers to a region where the new species was found.

#### Diagnosis.

Habitus similar to that of *Persanura hyrcanica*. Dorsal tubercles present and well developed. 3+3 pigmented eyes. Buccal cone short and relatively wide, labral sclerifications nonogival. Chaetotaxy of central area of head complete, chaetae A, B, C, D, E and O present. Tubercles Dl and (L+So) on head with six and ten chaetae respectively. Tubercles Di on Th. with two chaetae. Tubercles De on Th. II and III with five and six chaetae respectively. Tubercles De on Abd. I–III with four chaetae. Tubercles L on Abd. III and IV with five and eight chaetae respectively. Tubercles Di on Abd. V with three chaetae.

#### Description.

**Figures 14–28.**
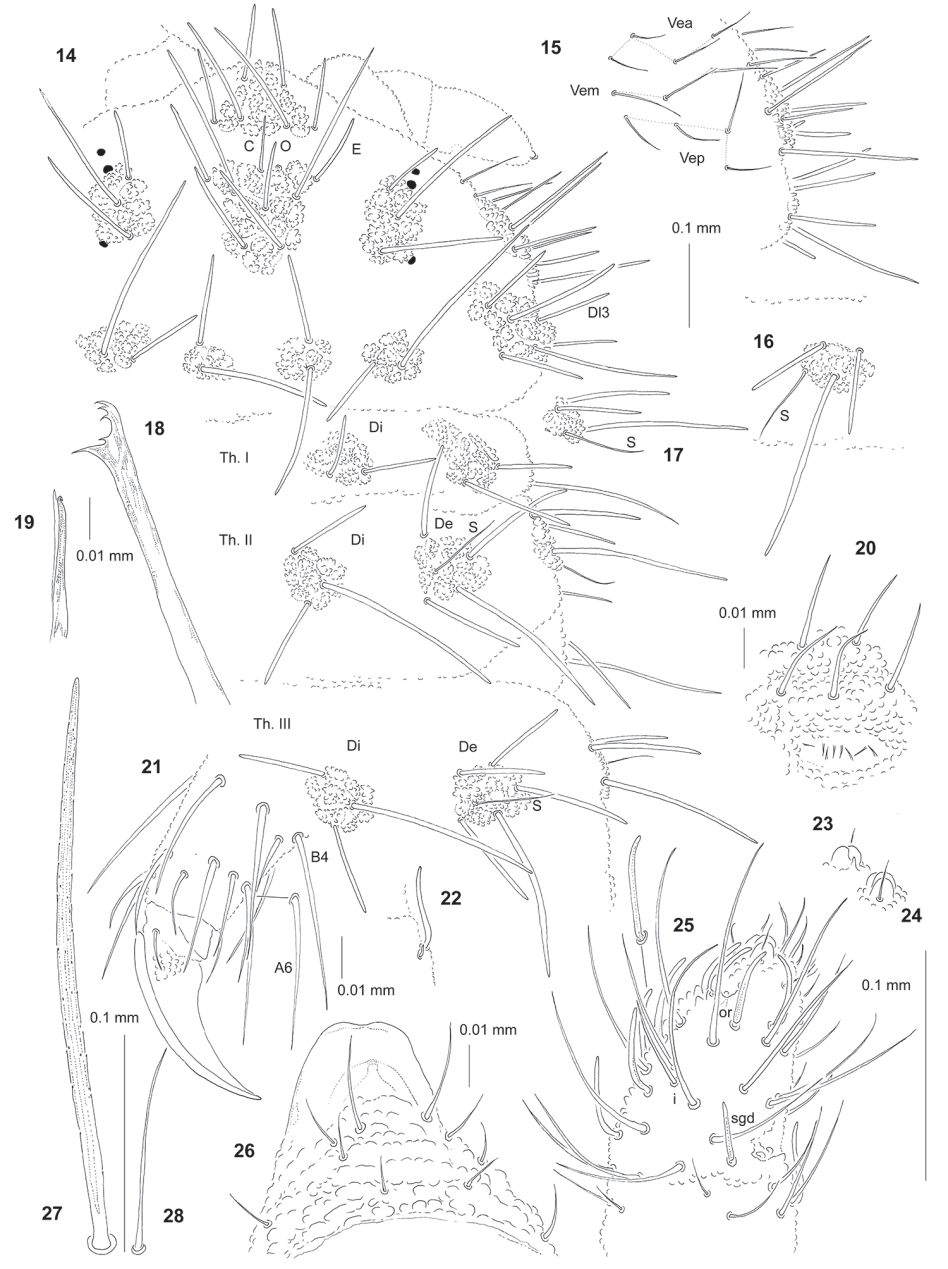
*Persanura lencarana* sp. n.: **14** chaetotaxy of head and Th. (holotype), dorsal view **15** chaetotaxy of ventrolateral part of head **16** tubercle De of Abd. II **17** tubercle Dl of Th. II, ventral view **18** mandible **19** maxilla **20** furca rudimentary **21** claw and T III, ventrolateral view **22** sensillum sgv and microsensillum of Ant. III **23** apical bulb, dorsal view **24** apical bulb, ventral view **25** dorsal chaetotaxy of Ant. III–IV **26** chaetotaxy and ventral sclerifications of labrum **27** chaeta Di1 of Abd. IV **28** sensillum of Abd. V.

General. Body length of holotype (without antennae): 2.15 mm. Colour of the body bluish. 3+3 small black eyes, in a typical arrangement for the genus (Fig. 14).

***Chaetal morphology.*** Dorsal ordinary chaetae of three types: long macrochaetae, short macrochaetae and mesochaetae. Long macrochaetae relatively thin, slightly arc-like or straight, narrowly sheathed, feebly serrated, apically rounded or pointed (Figs 14–17, 27). Macrochaetae Mc morphologically similar to long macrochaetae, but shorter. Mesochaetae similar to ventral chaetae, thin, smooth and pointed. S-chaetae of tergites thin, smooth and short, notably shorter than nearby macrochaetae (Figs 14, 16, 17, 28).

***Antennae.*** Typical of the genus. Dorsal chaetotaxy of Ant. III–IV as in Fig. 25 and Table 5. S-chaetae of Ant. IV relatively long and moderately thickened (Fig. 25). Apical vesicle well developed, trilobed (Figs 23, 24). Ventral chaetotaxy of Ant. III–IV as Table 5, sgv long and s-shaped (Fig. 22).

***Mouthparts.*** Buccal cone short and wide, with labral sclerifications nonogival. Labral chaetotaxy: 4/2, 4 (Fig. 26). Labium with four basal, three distal and four lateral chaetae, papillae x absent. Maxilla styliform, mandible strong with two basal and four apical teeth (Figs 18, 19).

***Dorsal chaetotaxy and tubercles.*** Chaetotaxy of head as in Figs 14, 15 and Table 4. Tubercle Cl with chaetae D. Chaetotaxy of thorax and abdomen as in Figs 14, 16, 17 and Table 6. Thorax and abdomen without free chaetae (Figs 14, 16). On Abd. I–III, the line of chaetae De1–chaeta s perpendicular to the dorsomedian line. Cryptopygy poorly developed, Abd. VI visible from above.

**Table 4.** Chaetotaxy of *Persanura lencarana* sp. n.: cephalic chaetotaxy, dorsal side.

Tubercle	Number of chaetae	Types of chaetae	Names of chaetae
Cl	6	Ml	F
		Mc	G, D
Af	8	Ml	A, B
		Mc	C, E, O
Oc	3	Ml	Ocm, Ocp
		Mc	Oca
Di	2	Ml	Di1
		Mc	Di2
De	2	Ml	De1
		Mc	De2
Dl	6	Ml	Dl5, Dl1
		Mc	Dl2–4, Dl6
(L+So)	10	Ml	L1, L4, So1
		Mc	L2, L3, So2, So6
		me	So3–5

**Table 5.** Chaetotaxy of *Persanura lencarana* sp. n.: chaetotaxy of antennae.

Segment, Group	Number of chaetae	Segment, Group	Number of chaetae adult
I	8	IV	or, 8 S, i, 12 mou, 6 brs, 2 iv
II	12		
III	5 sensilla AO III		
ve	5	ap	8 bs, 5 miA
vc	4	ca	2 bs, 3 miA
vi	4	cm	3 bs, 1 miA
d	5	cp	8 miA, 1 brs

**Table 6.** Chaetotaxy of *Persanura lencarana* sp. n.: postcephalic chaetotaxy.

Terga					Legs				
	Di	De	Dl	L	Scx2	Cx	Tr	Fe	T
Th. I	2	2	1	-	0	3	6	13	19
Th. II	3	4+s	3+s+ms	3	2	7	6	12	19
Th. III	3	5+s	3+s	3	2	8	6	11	18
					Sterna				
Abd. I	2	3+s	2	3	VT: 4				
Abd. II	2	3+s	2	3	Ve: 6; chaeta Ve1 present				
Abd. III	2	3+s	2	5	Vel:5–6; Fu: 5 me, 8 mi				
Abd. IV	2	2+s	3	8	Vel: 4–5; Vec: 2; Vei: 2; Vl: 4				
Abd. V	3	7+s			Ag: 3; Vl: 1, L‘: 1				
Abd. VI		7			Ve: 14; An: 2mi				

***Ventral chaetotaxy.*** On head, groups Vea, Vem and Vep with 4, 3, 4 chaetae respectively (Fig. 15). Group Vi on head with 6 chaetae. On Abd. IV, furca rudimentary with 8 microchaetae devoid of visible chaetopores (Fig. 20). On Abd. V, chaetae Vl and L’ present.

***Legs.*** Chaetotaxy of legs as in Table 6. Claw without internal tooth. On tibiotarsus, chaeta M present and chaetae B4 and B5 relatively short and pointed, chaeta A6 similarly in length to chaeta B4 (Fig. 21).

#### Remarks.

*Persanura lencarana* sp. n. most visibly differs from *P. hyrcanica* in the presence of a complete chaetotaxy in the central area of the head (reduced chaetae C, E and O absent in *hyrcanica*), the presence of two chaetae Di on Th. I (one chaeta in *hyrcanica*), the presence of three chaetae Di on Th. II–III (two chaetae in *hyrcanica*), the presence of four and five ordinary chaetae De on Th. II and III, respectively (two chaetae in *hyrcanica*), the presence of three ordinary chaetae De on Abd. I–III (two chaetae in *hyrcanica*), and the presence of three chaetae Di on the penultimate abdominal segment (two chaetae in *hyrcanica*). In addition, they differ in the number of labral chaetae (4/2, 4 in *lencarana*; 0/0, 4 in *hyrcanica*), the presence/absence of chaetae Dl3 on the head (present in *lencarana*; absent in *hyrcanica*), the number of chaetae L of Abd. IV (8 in *lencarana*; 3–5 in *hyrcanica*), and the presence/absence of microchaetae on furca rudimentary (present in *lencarana*; absent in *hyrcanica*).

## Discussion

The discovery of new species, e.g. *Albanura secunda* sp. n. and *Persanura lencarana* sp. n. described herein, in so far monotypic genera undoubtedly enriches and extends their characteristics providing new facts about their morphological differentiation. In some cases, nevertheless, they may reduce the number of distinguishing characters available for the genera. For example, the number and arrangement of labral chaetae in *P. lencarana* sp. n. is typical within the tribe and significantly different from the number described in the type species, *P. hyrcanica* Mayvan et al., 2015, where it was used as a one of the important generic feature of *Persanura* Mayvan et al., 2015 (Mayvan et al. 2015). On the other hand, strong mandibles, which were not used by the authors of this genus for comparison with other morphologically similar genera, like *Kalanura* Smolis, 2007, *Neanura* MacGillivray, 1893, and *Xylanura* Smolis, 2011 (MacGillivray 1893, Smolis 2007, 2011), appear to be a good and distinctive element of its characteristics. Likewise, *A. secunda* sp. n. is characterized by 3 + 3 chaetae Di on Abd. V as opposed to 2 + 2 chaetae in the type species of *Albanura* Deharveng, 1982. Thus, this feature cannot be further used as unique and diagnostic. Besides, new data on the morphological characteristics of originally monobasic genera, provide additional information that may support their recognition, such as geographic location. Interestingly, in the case of *Albanura*, a taxon morphologically closely related to *Deutonura* Cassagnau, 1979, but geographically separated, the author of the genus (Deharveng 1982) recognized the geographical criterion as important for its establishment. In the light of the discovery of *A. secunda* sp. n., the author’s decision seems to be justified as the distributional pattern of the genus is completely different from that which is known in *Deutonura*. Interestingly none species of *Deutonura* is known from the Caucasus where other genera of Neanurini, more or less widely distributed in the Western Palearctic, were observed e.g. *Endonura*, *Persanura* and *Caucasanura* Kuznetsova & Potapov, 1988. This last genus up to date includes two species, *Caucasanura stebayevae* Kuznetsova & Potapov, 1988, known from the Caucasus (Kuznetsova and Potapov 1988) and *Caucasanura besucheti* Deharveng, 1989, from north-eastern Turkey (Deharveng 1989).

In addition to supporting taxonomic decisions such as the establishing of monobasic genera, the species described in this work shed light on the origin and composition of the Neanurinae fauna of the Caucasus. The fauna of this subfamily in the region, despite our still poor knowledge, seems to contain elements belonging to the eastern part of Mediterranean Sea and Asia Minor as well as to the mountains in Iran. This assumption is consistent with recent observations on the genus *Endonura* from the Caucasus, where some of its representatives closely resemble both Mediterranean and Iranian species (Smolis et al. 2016a, b). Undoubtedly, further studies are necessary to understand the diversity and history of the subfamily in this extremely rich natural region, one of the two biodiversity hotspots located in the Western Palaearctic (Myers et al. 2000).

## Supplementary Material

XML Treatment for Albanura secunda

XML Treatment for Persanura lencarana
